# The Effect of Electrolytes on Blood Pressure: A Brief Summary of Meta-Analyses

**DOI:** 10.3390/nu11061362

**Published:** 2019-06-17

**Authors:** Sehar Iqbal, Norbert Klammer, Cem Ekmekcioglu

**Affiliations:** Department of Environmental Health, Center for Public Health, Medical University Vienna, Kinderspitalgasse 15, 1090 Vienna, Austria; n1637166@students.meduniwien.ac.at (S.I.); norbert.klammer@meduniwien.ac.at (N.K.)

**Keywords:** sodium, potassium, calcium, magnesium, electrolytes, blood pressure, hypertension, meta-analysis

## Abstract

Nutrition is known to exert an undeniable impact on blood pressure with especially salt (sodium chloride), but also potassium, playing a prominent role. The aim of this review was to summarize meta-analyses studying the effect of different electrolytes on blood pressure or risk for hypertension, respectively. Overall, 32 meta-analyses evaluating the effect of sodium, potassium, calcium and magnesium on human blood pressure or hypertension risk were included after literature search. Most of the meta-analyses showed beneficial blood pressure lowering effects with the extent of systolic blood pressure reduction ranging between −0.7 (95% confidence interval: −2.6 to 1.2) to −8.9 (−14.1 to −3.7) mmHg for sodium/salt reduction, −3.5 (−5.2 to −1.8) to −9.5 (−10.8 to −8.1) mmHg for potassium, and −0.2 (−0.4 to −0.03) to −18.7 (−22.5 to −15.0) mmHg for magnesium. The range for diastolic blood pressure reduction was 0.03 (−0.4 to 0.4) to −5.9 (−9.7 to −2.1) mmHg for sodium/salt reduction, −2 (−3.1 to −0.9) to −6.4 (−7.3 to −5.6) mmHg for potassium, and −0.3 (−0.5 to −0.03) to −10.9 (−13.1 to −8.7) mmHg for magnesium. Moreover, sufficient calcium intake was found to reduce the risk of gestational hypertension.

## 1. Introduction

Hypertension is the major leading risk factor for atherosclerosis and several diseases, especially renal and cardiovascular disorders, including myocardial infarction, stroke, and heart failure [1]. Blood pressure is influenced by various genetic and lifestyle factors including nutrition [2]. In this regard, sodium is an important mineral which, besides its functions in fluid balance, action potential generation, digestive secretions and absorption of many nutrients, also plays an important role in blood pressure regulation with a reduced sodium intake being associated with a reduction in systolic and diastolic blood pressure [3]. Therefore, independent of body weight, sex and age, too much dietary salt (sodium chloride) is regarded as an established risk factor for hypertension [4]. Concomitant to sodium reduction, higher potassium intake or supplementation has also been repeatedly shown to reduce the blood pressure of especially hypertensive persons (reviewed in [5]). Therefore, the American Heart Association recently proposed a dietary potassium intake of 3500–5000 mg/day, in addition to the well-known advice to reduce the consumption of dietary sodium (<1500 mg/day or at least 1000 mg/day decrement) for adults with normal and elevated blood pressure [6]. In addition, the WHO recommends that sodium consumption should be less than 2000 mg (5 g of salt) and potassium intake at least 3510 mg for adults per day [7].

In addition to the blood pressure lowering effects of sodium reduction or higher potassium intake also in several studies and meta-analyses calcium supplementation has been shown to exert beneficial effects on the risk for gestational hypertension [8,9], especially in women with low dietary calcium intake. 

Furthermore, from the electrolytes, magnesium is also effective in reducing blood pressure, especially by acting as a natural calcium channel blocker, increasing nitric oxide levels and improving endothelial dysfunction [10,11].

Early studies from the 1980s suggest that also chloride has an independent effect on blood pressure (reviewed in [12]). In these studies it was shown that replacing chloride with bicarbonate, citrate or phosphate as the anion for sodium did not lead to increases in blood pressure in rats or humans compared to sodium chloride [13,14,15]. On the other hand, interestingly, lower serum chloride levels were associated with higher cardiovascular and all-cause mortality risk in epidemiological studies [16]. 

Sulphur enters the body primarily as a component of the amino acids cysteine and methionine, and, as a part of the gaseous signaling molecule hydrogen sulfide (H_2_S), it exerts antihypertensive effects in experimental models [17]. Also garlic, which contains several functional sulfur-containing components, has been consistently shown to exert blood pressure-lowering effects with an average of 8–9 mmHg in systolic blood pressure (SBP) and 6–7 mmHg in diastolic blood pressure (DBP) in hypertensive patients [18]. 

Dietary phosphorus has also been shown to be related to blood pressure [19]. For example, in the International Study of Macro and Micro-Nutrients and Blood Pressure (INTERMAP) it was shown that dietary phosphorus was inversely associated with blood pressure in a multiple regression model [20]. Also in 13,444 participants from the Atherosclerosis Risk in Communities cohort and the Multi-Ethnic Study of Atherosclerosis cohorts compared with individuals in the lowest quintile of phosphorus intake at baseline, those with the highest phosphorus intake showed lower systolic and diastolic blood pressures after adjustment for dietary and non-dietary confounders [21]. 

Hypertension remains a serious public health issue and found as one amongst the major risk factors, also including smoking, high blood glucose, and high body-mass index, all responsible for approximately 29 million deaths globally [22]. According to the World Heart Federation, hypertension is the most important risk factor for stroke which causes about 50% of ischaemic strokes [23]. Nutrition has an important impact on blood pressure with salt playing a prominent role. However, also especially potassium, in addition probably magnesium, and calcium, and possibly also chloride, sulphur and phosphorus exert at least some effects on blood pressure. Therefore, the objective of this review was to summarize meta-analyses studying the effect and associations of these electrolytes as supplements or diets on human blood pressure or risk for hypertension throughout the last years.

## 2. Materials and Methods 

This review summarizes meta-analyses of publications studying the effect or association between electrolytes and blood pressure. In this regard, we conducted a search in PubMed, Scopus, and Google scholar databases by entering the search terms “(hypertension or blood pressure) and (sodium or potassium or calcium or magnesium or chloride or sulphur or sulphate or phosphorus or phosphate or salt) and (meta-analysis or metaanalysis)”. The literature search was conducted in June 2018 with a 10-year publication restriction in order to ensure more recent studies. Search results were limited to English language articles.

### 2.1. Eligibility Criteria

Meta-analyses of randomized controlled trials or observational studies were included in this review. The availability of (mean) blood pressure reductions and/or relative risk estimates e.g., risk ratios, odds ratios, weighted mean difference and confidence intervals were a prerequisite for the inclusion of the meta-analyses. Reviews and summaries of meta-analyses, meta-analysis not including the primary outcome, (e.g., blood pressure reduction or hypertension risk), or meta-analysis with combined effects of two minerals were excluded.

**Figure 1:**
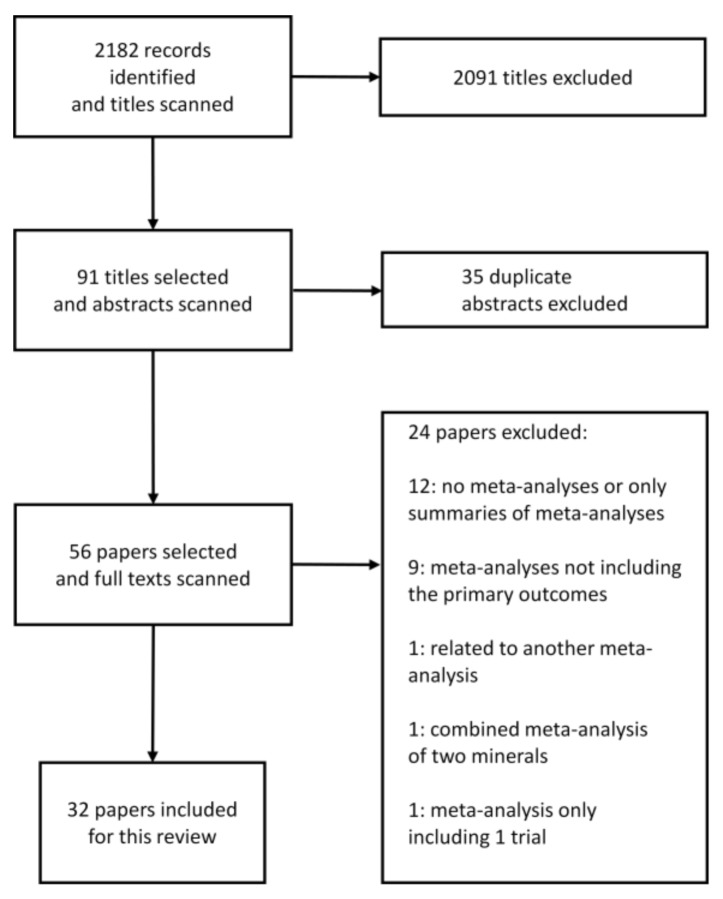
Flow chart of literature search to identify meta-analyses evaluating the effect of electrolytes on blood pressure or hypertension risk.

The initial search revealed a total of 2182 articles (Figure 1). After screening irrelevant, duplicate and other studies not meeting the inclusion criteria 32 meta-analyses were included in this review [8,11,24,25,26,27,28,29,30,31,32,33,34,35,36,37,38,39,40,41,42,43,44,45,46,47,48,49,50,51,52,53].

### 2.2. Data Extraction

Two reviewers (C.E and N.K) independently extracted the information from the papers. A third reviewer (S.I) rechecked the data and discrepancies were resolved through consensus. Study outcomes with defined number of trials/participants, study type/aims, patient characteristics, average study duration, minerals dosage (diet/supplements) and dietary modification were extracted from the selected meta-analyses. Also (mean) change in systolic and diastolic blood pressure (SBP and DBP), or relative risks (RR), odds ratios or effect sizes with 95% CI were extracted as main outcomes measures from the papers. 

## 3. Results

We identified meta-analyses for sodium, potassium, calcium and magnesium. No meta-analyses were found for the electrolytes chloride, sulphur and phosphorus.

### 3.1. Effect of Dietary Sodium/Salt Intake/Reduction on Blood Pressure

Overall, fourteen meta-analyses of randomized control trials and observational studies analyzing the effect of sodium modification/reduction on blood pressure were selected [24,25,26,27,28,29,30,31,32,33,34,35,36,37]. 

The meta-analyses of randomized controlled studies (*n* = 12) included 5–177 numbers of trials with 12 to 23,858 participants (Table 1). The duration of trials varied between 4 days to 71 months with sodium intake being reduced from 1.2 to 5.7 g/day (52–250 mmol/day) and salt reduction was 2 to 9.6 g/day (34−164 mmol/day). The results showed that blood pressure lowering effects ranged from −0.7 (95% confidence interval: −2.6 to 1.2) mmHg for lowest and −8.9 (−14.1 to −3.7) mmHg for highest SBP reduction, respectively, while lowest to highest reduction for DBP was between 0.03 (−0.4 to 0.4) to −5.9 (−9.7 to −2.1) mmHg.

Moreover, two meta-analyses included observational studies, including 10–18 trials with 8093–134,916 participants. The observational study of Talukder et al. [36] observed a mean difference in blood pressure of 0.1 (−0.2 to 0.3) mmHg for SBP and 0.2 (0.1 to 0.4) mmHg for DBP with exposure of 4–405 mg/L water sodium levels. Subasinghe et al. [37] showed effect sizes of 1.36 (1.24 to 1.48) and 1.28 (1.13 to 1.45) for high salt exposure (6.9 to 42.3 g/day) on hypertension risk in rural and urban populations of low-and-middle income countries, respectively.

### 3.2. Effect of Potassium Supplementation on Blood Pressure

Five meta-analyses of randomized controlled trials evaluated the effect of oral potassium supplements on blood pressure [38,39,40,41,42]. The meta-analyses included 10–33 trials and 556–1892 participants (Table 2). Oral potassium dosages in the supplements were between 6 and 250 mmol/day (0.23–9.7 g/day) with study durations of 4–52 weeks. The lowest to highest reduction in blood pressure was between –3.5 (95% confidence interval: −5.2 to −1.8) to −9.5 (−10.8 to −8.1) mmHg for SBP and −2 (−3.1 to −0.9) to −6.4 (−7.3 to −5.6) mmHg for DBP. In addition, potassium was found to be especially effective in in reducing blood pressure of high sodium consumers.

### 3.3. Calcium Intake in Form of Supplements or Diets and Risk for Gestational Hypertension or Blood Pressure Lowering

We identified six meta-analyses which evaluated the association of dietary calcium intake on the risk for gestational hypertension [8,43,44,45] or the effect on blood pressure [46,47]. Five meta-analyses of randomized controlled trials with a range of 4–16 trials and 2947–36,806 participants were included. The follow up intervention period was between 8 weeks to 7 years and the calcium intake was found to be between 0.5 g/day to 2 g/day. The results showed RR ranging from 0.55 to 0.91 for gestational hypertension while, the meta-analysis of Cormick et al., [47] found a mean difference of −1.4 (95% confidence interval: −2.2 to −0.7) mmHg for SBP and −1 (−1.5 to −0.5) mmHg for DBP reduction in normotensive people.

Furthermore, we found one meta-analysis including 16 observational trials of 757–41,214 pregnant women showing a lower OR for gestational hypertension [OR: 0.63 (95% CI = 0.41–0.97)] for highest versus lowest category of calcium intake [45].

Most of the meta-analyses showed that calcium supplements were associated with a reduced risk of gestational hypertension (Table 3). Besides, based on the results from the meta-analysis of Wu and Sun [46] calcium plus vitamin D supplementation non-significantly slightly increased SBP with no effects on DBP. 

### 3.4. Effect of Magnesium on Blood Pressure or Hypertension Risk

Eight meta-analyses of randomized control trials (*n* = 5) and observational studies (*n* = 3) were included to summarize the effects of magnesium on blood pressure or association with hypertension risk, respectively [11,45,48,49,50,51,52,53] The randomized control trials included 7–28 number trials with 135–1694 of participants with the trial durations varying between 3–24 weeks. Magnesium intake ranged between 120–1006 mg/day. The summary showed SBP reductions in the range of −0.2 (95% confidence interval: −0.4 to −0.03) mmHg and −18.7 (−22.5 to −15.0) mmHg, and DBP reductions between −0.3 (−0.5 to −0.03) and −10.9 (−13.1 to −8.7) mmHg (Table 4). However, the meta-analysis of Rosanoff and Plesset (2013) [51], which showed the largest effects, only included a small sample of treated hypertensive patients, which probably responded highly to magnesium. When omitting this meta-analysis, the blood pressure lowering effects of magnesium would switch to a rather low to moderate level.

Moreover, observational studies showed a lower risk for hypertension with increasing magnesium intake [52] or higher circulating magnesium levels [53], respectively.

## 4. Discussion

The major findings of this review were especially that sodium (salt) reduction and a higher intake of potassium have convincing blood pressure lowering effects. In addition, higher magnesium intake is suggested to possibly reduce blood pressure, especially in patients with hypertension. Moreover, sufficient calcium intake confers a protective effect regarding the risk for gestational hypertension. 

There are several mechanistical explanations for the association between high sodium intake and blood pressure like enhanced reabsorption and retention of filtered sodium through the renal tubules [54], or activation of the brain renin–angiotensin–aldosterone system (RAAS), which is suggested to increase blood pressure through angiotensin II and aldosterone promoting locally oxidative stress and activating the sympathetic nervous system [55]. Furthermore, based on the “vasodysfunction theory” of salt induced hypertension, salt loading results in subnormal decreases in systemic vascular resistance leading to an increase in blood pressure [56]. In this regard salt sensitivity, which varies among individuals, is suggested to play an important role [3,55].

In addition to blood pressure, in a recent systematic review and meta-analysis of randomized controlled trials it was analyzed that restriction of dietary sodium intake can reduce arterial stiffness, as expressed by carotid-femoral pulse wave velocity [57].

In addition to sodium modification, several studies and meta-analysis showed that potassium supplements reduce blood pressure and also the risk of hypertension [58]. Suggested mechanisms of the blood pressure lowering effects of potassium are (reviewed in [5]): improvement of endothelial function and NO release, vasodilatation by lowering cytosolic smooth muscle cell calcium, increasing natriuresis, and lowering the activity of the sympathetic nervous system. Furthermore the supportive blood pressure reducing effects of sodium modification in combination with potassium is well-documented [59]. 

Besides the interdependent effect of sodium and potassium, calcium and magnesium have also been implicated in the regulation of blood pressure [60]. Based on our results, magnesium showed a moderate blood pressure reducing effect in general, while the effects of calcium were primarily restricted to the prevention of gestational hypertension. The potential antihypertensive effects of magnesium are for example suggested to be related to calcium channel blockage, increases in nitric oxide, and better endothelial function [10]. In vascular smooth muscle cells, magnesium antagonizes Ca^2+^ by inhibiting transmembrane calcium transport and calcium entry with low magnesium levels causing an increase in intracellular free Ca^2+^ concentration and subsequently vascular contraction [61]. 

On the other hand, low dietary calcium intake is shown to be a risk factor for the development of hypertension especially for women with a history of gestational hypertension [62]. In this regard the World Health Organization recommends daily calcium supplementation of 1.5–2.0 g oral elemental calcium for pregnant women in populations with low dietary calcium intake to reduce the risk of pre-eclampsia and related complications [63]. 

Lifestyle modifications including weight loss, exercise, and healthy diets are proved to be important predictors to lower blood pressure or the risk for hypertension, respectively. For example, the Canadian hypertension education program guidelines (2016) proposed health behavior management for the prevention of hypertension such as physical exercise, weight management, limited alcohol consumption, stress management, reduce sodium intake and recommended dietary modification as the preferred method of increasing potassium intake (in patients who are not at risk of hyperkalemia) to get additional nutritional benefits of whole foods over prescribed supplements [64]. Similarly, the study findings of a systematic review and meta-analysis of randomized control trials showed that healthy dietary patterns including the dietary approaches to stop hypertension (DASH) diet, Nordic diet, and Mediterranean diet significantly lowered systolic and diastolic blood pressure [65].

## 5. Conclusions and Future Perspectives

Our brief exploration of meta-analyses showed that lowering sodium and increasing potassium intake would exert convincing blood pressure lowering effects, especially in hypertensive patients. The maximum extent of systolic blood pressure lowering was approximately in the order of 8 to 9 mmHg, which roughly equals a monotherapy with an antihypertensive drug [66]. This reflects the significant importance of healthy nutrition, in this case salt reduction and increase of dietary potassium intake, on blood pressure. Our summary of meta-analytic reviews also suggests that higher magnesium intake may exert beneficial effects on blood pressure, although the results were rather moderate. However, in some (treated) hypertensive patients (“high-responders”) higher magnesium intake might result in larger effects. So, increasing/optimizing dietary magnesium intake could also be a helpful recommendation in patients with hypertension. Furthermore, there is convincing evidence that increasing/optimizing calcium intake can lower the risk for gestational hypertension, especially in women with initial low calcium intake. 

We did not find any meta-analyses summarizing the effects of chloride, phosphorus and sulphur on blood pressure or hypertension risk. In general, few studies are available, which assessed the effect of these electrolytes on blood pressure. There are indications from observational studies that there might be some effects, however robust evidence is missing. For example, a very recent systematic review from McClure et al. (2019) did not detected a consistent association between total dietary phosphorus intake and blood pressure [67].

Relating to earlier studies replacing the chloride component of salt with other anions like bicarbonate or citrate might be a certain strategy, which could not only beneficially affect blood pressure but could show co-benefits regarding a (western) diet induced metabolic acid load [68]. Obviously, randomized (controlled) trials are necessary to study this assumption. Also, future clinical trials could determine the effect of different electrolytes on blood pressure by patients’ self-assessment of blood pressure with new validated mHealth devices, like a previous study in obese individuals has shown [69]. 

## Figures and Tables

**Table 1:** Effect of dietary sodium/salt reduction on blood pressure: A summary of meta-analyses of randomized (controlled) trials or observational studies.

Author/Year	No. of Trials	Study Characteristics	No. of Participants	Patient Characteristics	Duration of Trials	Sodium/Salt Intake or Reduction	Blood Pressure Lowering in mmHg (95% CI)	Further Remarks/Summary
Aburto et al., 2013a [24]	36	Randomized controlled trials	6736	2273 (hypertensive)	Most studies (*n* = 31) <3 months	Different reductions in sodium intake Relative sodium reduction in the intervention group: ≥1/3 of control	SBP: −3.4 (−4.3 to −2.5) DBP: −1.5 (−2.1 to −1.0)	Reduced sodium intake decreases blood pressure in people both with and without hypertension. The reduction in blood pressure was greater in those with hypertension.
Adler et al., 2014 [25]	6 (SBP) 5 (DBP)	Randomized controlled trials	3362 (SBP) 2754 (DBP)	SBP (end of trial): (normotensive) 2079 (hypertensive) 1283 DBP (end of trial): (normotensive) 2079 (hypertensive) 675	7–36 months	Sodium intake: 70 to >100 mmol/day	Normotensive: SBP: −1.2 (−2.3 to 0.02) DBP: −0.8 (−1.4 to −0.2) Hypertensive: SBP: −4.1 (−5.8 to −2.4) DBP: −3.7 (−8.4 to 0.9)	Normotensive persons: small blood pressure reduction. Hypertensive patients: greater reduction in SBP, no difference in DBP.
Graudal et al., 2015 [26]	15	Randomized controlled trials	12–114	“time to maximal efficacy” analysis 7 studies with hypertensive patients 7 studies with normotensive persons 1 study hypertensive + normotensive	1 to 6 weeks	Sodium reduction range: 55–118 mmol/day	No significant differences in SBP or DBP after initiation of salt reduction between week 1 and subsequent weeks.	Time dependent effects of salt reduction on blood pressure. The effect of salt reduction on blood pressure appears to reach maximal efficacy at 1 week and remain stable over subsequent time intervals.
Graudal and Jürgens 2015 [27]	92	Randomized controlled trials	661 Asians 561 Blacks 3782 Whites	9 Asian/9 Black/74 White population	7–365 days	Sodium reduction: 63–103 mmol	SBP: −3.2 (−4.0 to −2.5) (in Whites) −4.7 (−7.1 to −2.3) (in Blacks) −3.8 (−6.4 to −1.3) (in Asians) DBP:−1.5 (−2.1 to −1.0) (in Whites) −3.0 (−4.0 to −2.0) (in Blacks) −2.0 (−3.0 to −0.9) (in Asians)	SBP: no differences in ethnic groups. DBP: small differences between black and white people.
Gay et al., 2016 [28]	24	Randomized controlled trials	23,858	11 to 2570 participants (median: 129) participants >19 years old	Trial durations ranged from 6 to 48 months of follow−up (median: 12 months)	Dietary interventions (including low sodium diets)	Overall pooled net effect of diets: SBP: −3.1 (−3.9 to −2.3) DBP: −1.8 (−2.2 to −1.4)	This meta-analysis shows that dietary interventions (including low sodium diets) provide clinically significant net blood pressure reductions, and that some dietary patterns may be more effective than others.
Graudal et al., 2017 [29]	177	Randomized controlled trials	12,210	White people with hypertension. (84 studies; 5925 participants in SBP; 85 studies; 6001 participants in DBP) Black people with hypertension. (8 studies; 619 participants in SBP and DBP) Asian people with hypertension. (8 studies; 501 participants in SBP and DBP) White people with normotension. (89 studies, 8569 participants in SBP; 90 studies, 8833 participants in DBP) Black people with normotension. (7 studies, 506 participants in SBP and DBP) Asian people with normotension. (3 studies, 393 participants in SBP and DBP)	4–1100 days	Mean sodium reduction: 135 mmol/day range; <100 to ≥250 mmol/day	Hypertensive (White): SBP: −5.5 (−6.5 to −4.6) DBP: −2.9 (−3.4 to −2.3) Hypertensive (Black): SBP: −6.6 (−9.0 to −4.2) DBP: −2.9 (−4.5 to −1.30) Hypertensive (Asian): SBP: −7.8 (−11.4 to −4.1) DBP: −2.7 (−4.2 to −1.2) Normotensive (White): SBP: −1.1 (−1.6 to −0.6) DBP: 0.03 (−0.4 to 0.4) Normotensive (Black) SBP: −4.0 (−7.4 to −0.7) DBP: −2.0 (−4.4 to 0.4) Normotensive (Asian): SBP: −0.7 (−3.9 to 2.4) DBP: −1.6 (−3.4 to 0.1)	High-quality evidence for White people; moderate-quality evidence for Black/Asian people
He et al., 2013 [30]	34	Randomized trials	3230	990 (of 22 trials) hypertensive 2240 (of 12 trials) normotensive	Median duration: 5 weeks in hypertensive people, 4 weeks in normotensive people	Salt reduction: 75 mmol/day (4.4 g/day). Reduction of urinary sodium: 40–120 mmol/day (2.3–7.0 g/day).	Total SBP: −4.2 (−5.2 to −3.2) DBP: −2.1 (−2.7 to −1.5) Hypertensive: SBP: −5.4 (−6.6 to −4.2) DBP: −2.8 (−3.5 to −2.1) Normotensive: SBP: −2.4 (−3.6 to −1.3) DBP: −1.0 (−1.9 to −0.2)	Reduction in SBP was significant in both black and white people and in women and men. Significant effects on blood pressure were seen in hypertensives and normotensives. Dose-response relation: the greater the reduction in salt intake, the greater the fall in blood pressure.
He and MacGregor 2011 [31]	6	Outcome trials	6250	3 trials in normotensive participants 3 trials in hypertensive patients	6–36 months	Salt reduction: 2–2.3 g/day	Normotensive: SBP: −1.1 (−0.1 to 2.3) DBP: −0.8 (0.2 to 1.4) Hypertensive: SBP: −4.1 (2.4 to 5.8) DBP: −3.7 (−0.9 to 8.4)	Significant reduction in cardiovascular events
Kelly et al., 2016 [32]	5	Randomized and non-randomized controlled trials	1214	Normotensive participants (≥18 years) with SBP ≤140 mmHg	4 weeks to 48 months	Salt reduction: −75 mmol/day (range; −37 to −136 mmol).	SBP: −0.7 (−2.6 to 1.2) DBP: −0.6 (−1.3 to 0.1)	No significant change in SBP or DBP following reduction of dietary sodium over the period of 4 weeks to 36 months
Peng et al., 2014 [33]	5	Randomized controlled trials	1974	Hypertensive and normotensive participants	6 months to 2 years	Different salt substitutes vs. common salt (NaCl).	SBP: −4.9 (−7.3 to −2.5) DBP: −1.5 (−2.7 to −0.3)	Salt substitutes significantly reduced both SBP and DBP
Taylor et al., 2011 [34]	7	Randomized controlled trials	3 trials normotensive (3518), 2 trials hypertensive (758), 1 trial mixed pop. (1981), 1 trial with heart failure (234)	Adults ≥18 years, irrespective of gender/ethnicity. Studies of children/pregnant women were excluded.	Trials follow-up ranged 6 to 71 months	Salt reduction; <70–100 mmol/ day. Urinary 24-h sodium excretion: Normotensive (mean diff.): 34.2 mmol/24 h (18.8–49.6), Hypertensive (mean diff.): 39.1 mmol/24 h (31.1–47.1)	Normotensives (mean difference) SBP:−1.1 (−2.3 to 0.1) DBP:−0.8 (−1.4 to −0.2) Hypertensives SBP: −4.1 (−5.8 to −2.4) DBP: −3.7 (−8.4 to 0.9)	Significant reduction of SBP in hypertensive patients
Wang et al., 2015 [35]	6	Interventional studies	3153	Chinese adults aged ≥35 years	At most 1 week	Salt level reduced in hypertensive patients: 9.6 g/day (163.0 mmol/day sodium).	Normotensive + hypertensive: SBP: −6.3 (−7.2 to −5.4) DBP: −3.2 (−3.7 to −2.7) Hypertensive: SBP: −8.9 (−14.1 to −3.7) DBP: −5.9 (−9.7 to −2.1)	Salt restriction lowers mean BP in Chinese adults, with the strongest effect among hypertensive participants.
**Observational Studies**								
Talukder et al., 2017 [36]	10	Observational studies	8093	7 studies (12 datasets) with 3747 participants with low/high water sodium exposure groups		4–405 mg/L water sodium level	Standardized mean difference: SBP: 0.1 (−0.2 to 0.3) DBP: 0.2 (0.1 to 0.4)	An (inconclusive) association between water sodium and human blood pressure is suggested, more consistently for DBP.
Subasinghe et al., 2016 [37]	18	Observational studies	134,916	Participants in urban and rural areas in low-and-middle income countries (LMICs). Age: 24–65.		Daily salt intake range: 6.9 to 42.3 g/day	Effect size (ES) of hypertension ES 1.36 (1.24 to 1.48) ES 1.28 (1.13 to 1.45)	Excessive salt intake has a greater impact on the prevalence of hypertension in urban than rural regions.

SBP = Systolic blood pressure; DBP = Diastolic blood pressure.

**Table 2:** Effect of potassium supplementation on blood pressure: A summary of meta-analyses of randomized controlled trials.

Author/Year	No. of trials	Study Characteristic	No. of Participants	Patient Characteristics	Duration of Trials	Potassium Dosage (Supplements)	Blood Pressure Lowering in mmHg (95%CI)	Further Remarks/Summary
Aburto et al., 2013b [38]	21	Randomized controlled trials	1892/1857	Hypertensive 818(SBP)/828(DBP)	<2 to >4 months	<90 mmol/day to >155 mmol/day in the intervention group	SBP: −3.5 (−5.2 to −1.8) DBP: −2.0 (−3.1 to −0.9)	Effect seen in people with hypertension but not in those without hypertension. Intake above 120 mmol/day did not seem to have any additional benefit. Potassium may be more effective in reducing blood pressure at higher levels of sodium consumption.
Binia et al., 2015 [39]	15	Randomized controlled trials	917	400 hypertensives 329 normotensives 188 hypertensive or normotensive persons (mixed population)	4–24 weeks	<40–120 mmol/day	All: SBP: −4.7 (−7.0 to −2.4) DBP: −3.5 (−5.7 to −1.3) Hypertensive patients: SBP: −6.8 (−9.3 to −4.3) DBP: −4.7 (−7.5 to −1.8)	Potassium supplementation is associated with reduction of blood pressure in patients who are not on antihypertensive medication, and the effect is significant in hypertensive patients.
Filippini et al., 2017 [40]	33	Randomized controlled trials	1829	1163 (studies ≥4 weeks overall)	<4 to ≥12 weeks	25–250 mmol/day	SBP: −4.5 (−5.9 to −3.1) DBP: −3.0 (−4.8 to −1.1)	Potassium supplementation in hypertensives was generally associated with decreased blood pressure, particularly in high sodium consumers.
Poorolajal et al., 2017 [41]	23	Randomized controlled trials	1213	Primary hypertension: 732 (SBP) 695 (DBP)	4–52 weeks	6–200 mmol/day	SBP: −4.3 (−6.0 to −2.5) DBP: −2.5 (−4.1 to −1.0)	Potassium supplementation has a modest but significant impact on blood pressure.
Bommel and Cleophas 2012 [42]	10	Crossover and parallel design studies	556	High salt intake, >170 mmol/24h	Follow up 8–16 weeks	Not available	SBP: −9.5 (−10.8 to −8.1) DBP: −6.4 (−7.3 to −5.6)	Potassium treatment reduces considerably the blood pressure of hypertensive patients on salt rich diets.

SBP = Systolic blood pressure; DBP = Diastolic blood pressure.

**Table 3:** Calcium intake in form of diets or supplements and risk for gestational hypertension or effect on blood pressure: a summary of meta-analyses of randomized controlled trials or observational studies.

Author/Year	No. of Trials	Study Characteristic	No. of Participants	Study Aims	Duration of Trials	Calcium Dosage (Diet or Supplement)	Blood Pressure Lowering in mmHg or RR/OR for Gestational Hypertension (95% CI)	Further Remarks/Summary
Imdad et al., 2011 [8]	6	Randomized controlled trials	Calcium-group: 4919 Control group: 4942	Effect of calcium supplementation on gestational hypertensive disorders in studies from developing countries	Calcium supplements in all the included studies were before 20–32 weeks of gestation and continued till delivery.	0.5–2 g/day	RR: 0.55 (0.36 to 0.85)	Calcium supplementation during pregnancy was associated with a significant reduced risk of acquiring gestational hypertension.
Hofmeyr et al., 2014 [43]	12 trials	Randomized controlled trials	15,470 women	Assessing the effects of calcium supplementation during pregnancy on hypertensive disorders of pregnancy and related maternal and child outcomes	Calcium supplementation started at the latest from 34 weeks of pregnancy.	High-dose calcium supplementation (≥1 g/day)	RR: 0.65 (0.53 to 0.81)	Average risk of high blood pressure was reduced with calcium supplementation compared with placebo. There was also a reduction in hypertension with low-dose calcium supplementation (<1 g/day).
An et al., 2015 [44]	4	Randomized controlled trials	Gestational hypertension: 7252 Control group: 7272 Severe gestational hypertension: 6673 Control group: 6684	Assessing the effectiveness of calcium supplementation during pregnancy on reducing the risk of hypertensive disorders of pregnancy and related problems.	From ~11–24 weeks of pregnancy to delivery	Supplementation with calcium (at least >1 g/day)	Gestational hypertension: RR: 0.91 (0.84 to 0.99) Severe gestational hypertension: RR: 0.81 (0.60 to 1.09)	Calcium supplementation appears to reduce the risk of hypertension in pregnancy. No significant reduction in the risk of severe gestational hypertension.
Wu and Sun 2017 [46]	8	Randomized controlled trials	36,806	Evaluation the effect of calcium plus vitamin-D (CaD) supplements on the changes in BP from baseline to the longest follow-up time point in male and female participants.	8 weeks to 7 years	Intervention dose of calcium (≤1000 mg/day, 5 trials or >1000 mg/day, 3 trials)	Mean differences in SBP: 0.6 (−1 to 2.20) Mean differences in DBP: −0.2 (−0.9 to 0.5)	Calcium plus vitamin D supplementation slightly increased SBP, but the difference was not statistically significant. Calcium plus vitamin D supplementation did not significantly affected DBP reduction.
Cormick et al., 2015 [47]	16	Randomized controlled trials	SBP: 3048 (16 studies) DBP: 2947 (15 studies)	Assessing the efficacy and safety of calcium supplementation versus placebo or control for reducing blood pressure in normotensive people	Median follow up intervention period of 3.5 months	For most studies the intervention was 1000 mg to 2000 mg of elemental calcium per day	Mean difference: SBP: −1.4 (−2.2 to −0.7) DBP: −1 (−1.5 to −0.5)	The quality of evidence was high for doses of calcium of 1000 to 1500 mg/day and was moderate for lower or higher doses. Calcium intake slightly reduced both SBP and DBP in normotensive people.
**Observational studies**								
Schoenaker et al., 2014 [45]	16	Observational studies	Case-control studies: 757 pregnant women Cohort studies: 41,214 pregnant women, 908 gestational hypertension	Assessing the effect of dietary factors on hypertensive disorders during pregnancy (gestational hypertension and pre-eclampsia)		Highest group >1600 mg/day versus lowest group <1000 mg/day	Gestational hypertension (comparing highest to lowest): OR: 0.63 (0.41 to 0.97)	Results from case–control studies consistently showed lower reported calcium intake for pregnant women with hypertensive disorders (gestational hypertension and preeclampsia)

SBP = Systolic blood pressure; DBP = Diastolic blood pressure.

**Table 4:** Effect magnesium on blood pressure or association with hypertension risk: A summary of meta-analyses of randomized controlled trials and observational studies.

Author-Year	No. of Trials	Study Characteristics	No. Participants	Study Aims	Duration of Trials	Magnesium Dosage (Diet or Supplement)	Blood Pressure Lowering in mmHg or RR (95% CI)	Further Remarks/Summary
Zhang et al., 2016 [11]	27	Randomized controlled trials	Magnesium group: 822 Placebo group: 800	Effect of magnesium supplementation in normotensive and hypertensive adults (age 18–84 years).	3 weeks–6 months	Median dose of 368 mg/day (range: 238–960 mg/day)	SBP: −2 (−0.4 to −3.6) DBP: −1.8 (−0.7 to –2.8)	Magnesium supplementation at a median dose of 368 mg/day for a median duration of 3 months significantly reduced SBP and DBP. Magnesium supplementation at a dose of 300 mg/day or duration of 1 month is enough to elevate serum magnesium and reduce blood pressure. Serum magnesium was negatively associated with DBP but not SBP.
Dibaba et al., 2017 [48]	11	Randomized controlled trials	543	Assessing the pooled effect of magnesium supplementation on blood pressure in participants with preclinical or non−communicable diseases.	1 to 6 months (mean: 3.6 months)	365–450 mg/day	Standardized mean difference: SBP: −0.2 (−0.4 to −0.03) DBP: −0.3 (−0.5 to −0.03)	Magnesium supplementation lowers blood pressure in individuals with insulin resistance, prediabetes, or other noncommunicable chronic diseases.
Verma and Garg 2017 [49]	28	Randomized controlled trials	1694 (834 treatment arm, 860 placebo arm)	Evaluation the effect of magnesium supplementation on type 2 diabetes associated cardiovascular risk factors in both diabetic and nondiabetic individuals. Only four studies were carried out in hypertensive subjects.	4−24 weeks	Elemental magnesium: 300–1006 mg/day	Weighted mean difference: SBP: −3.06 (−5.51 to −0.60) DBP: −1.37 (−3.02 to 0.29)	A significant improvement was observed in SBP. Insignificant improvement or no improvement was observed in DBP
Kass et al., 2012 [50]	22	Interventional studies	1173	Assessing the effect of magnesium supplementation on blood pressure. Adults from 12 different countries were included.	3 to 24 weeks of follow-up	Elemental magnesium dosage: 120–973 mg/day	Overall effect size: SBP: 0.3 (0.2 to 0.4) DBP: 0.4 (0.3 to 0.4)	Summary of all trials show a decrease in SBP of 3–4 mmHg and DBP of 2–3 mmHg. Magnesium supplementation appears to achieve a small but clinically significant reduction in blood pressure.
Rosanoff and Plesset 2013 [51]	7	Interventional studies	135 treated hypertensive subjects	Evaluation of magnesium supplementation in hypertension. Initial SBP of the patients was >155 mmHg	6 to 17 weeks	10.5–18.5 mmol magnesium-salt/day	Mean change: SBP: −18.7 (−22.5 to −15.0) DBP: −10.9 (−13.1 to −8.7)	This uniform subset of seven studies showed a strong effect of magnesium in treated hypertensive patients.
**Observational studies**								
Schoenaker et al., 2014 [45]	3	Observational studies	6616 pregnant women, age range 20–40 years	Assessing the effect of dietary factors, including magnesium, on hypertensive disorders of pregnant women.	NA	Not indicated	Significantly lower mean magnesium intake of mean 7.69 mg/day for women with hypertensive disorders of pregnancy (gestational hypertension and pre-eclampsia)	Pooled results revealed statistically significantly lower mean magnesium intake for women with hypertensive disorders of pregnancy.
Han et al., 2017 [52]	10	Prospective cohort studies	180,566 participates	Assessing the relationship between dietary magnesium intake and serum magnesium concentrations on the risk of hypertension in adults. Adult population >18 years was included.	4–15 years	96–425 mg/day	RR: 0.95 (0.90 to 1.00) for a 100 mg/increment in magnesium intake. Comparing highest to lowest: RR: 0.91 (0.80 to 1.02)	Increase in magnesium intake was associated with a lower risk of hypertension in a linear dose-response pattern.
Wu J et al., 2017 [53]	3	Prospective cohort studies with four cohorts	14,876 participants (3149 cases)	Evaluation of circulating magnesium levels and incidence of coronary heart diseases, hypertension, and type 2 diabetes mellitus	Average of 6.7 years of follow-up	NA	Per 0.1 mmol/L increment in serum magnesium levels: RR: 0.96 (0.93 to 0.99)	A significant inverse linear association was observed between circulating magnesium levels and incidence of hypertension.

SBP = Systolic blood pressure; DBP = Diastolic blood pressure; NA = not applicable.
